# Swept-source optical coherence tomography angiography of diabetic papillopathy: a case report

**DOI:** 10.1186/s12886-020-01470-5

**Published:** 2020-05-15

**Authors:** Ji Min Choi, Hye Jin Lee, Dae Joong Ma

**Affiliations:** 1 Department of Internal Medicine, Healthcare Research Institute, Seoul National University Hospital Healthcare System Gangnam Center, Seoul, Republic of Korea; 2 Department of Ophthalmology, Jeju National University College of Medicine, Jeju-si, Jeju-do Republic of Korea; 3 Department of Ophthalmology, Hallym University Kangnam Sacred Heart Hospital, 1, Singil-ro, Yeongdeungpo-gu, Seoul, 07441 Republic of Korea

**Keywords:** Diabetic Papillopathy, Swept-source optical coherence tomography angiography, Disc neovascularization

## Abstract

**Background:**

We report a case of diabetic papillopathy (DP) diagnosed using swept-source optical coherence tomography angiography (SS-OCTA).

**Case presentation:**

A 52-year-old man was referred for evaluation of a swollen optic disc in both eyes. His best-corrected visual acuity was 20/40 in the right eye and 20/100 in the left eye. Fundus examination demonstrated a swollen optic disc, splinter hemorrhages, and radially oriented, dilated vessels over the optic disc in both eyes. Laboratory tests revealed previously unknown diabetes. SS-OCTA was performed to rule out neovascularization of the disc (NVD). B-scan image displayed blood flow signals in the thickened retinal nerve fiber layer of the optic disc and not above the vitreoretinal interface. We diagnosed the patient with DP.

**Conclusions:**

This case showed that SS-OCTA is useful for distinguishing DP from NVD.

## Background

Diabetic papillopathy (DP) is defined as unilateral or bilateral optic disc swelling with no detectable etiology other than diabetes mellitus (DM) [1]. DP is self-limiting and usually resolves over a period of 2–10 months, without significant visual sequelae [1]. Currently, DP it is diagnosed by excluding the other causes of disc swelling, including inflammation, ischemia, infiltration, direct optic nerve compression, trauma, toxicity, and increased intracranial pressure [2]. Disc vessel changes with fine telangiectatic vessels are accompanied in DP, which can be misdiagnosed as neovascularization of the disc (NVD) [1, 3].

**Fig. 1:**
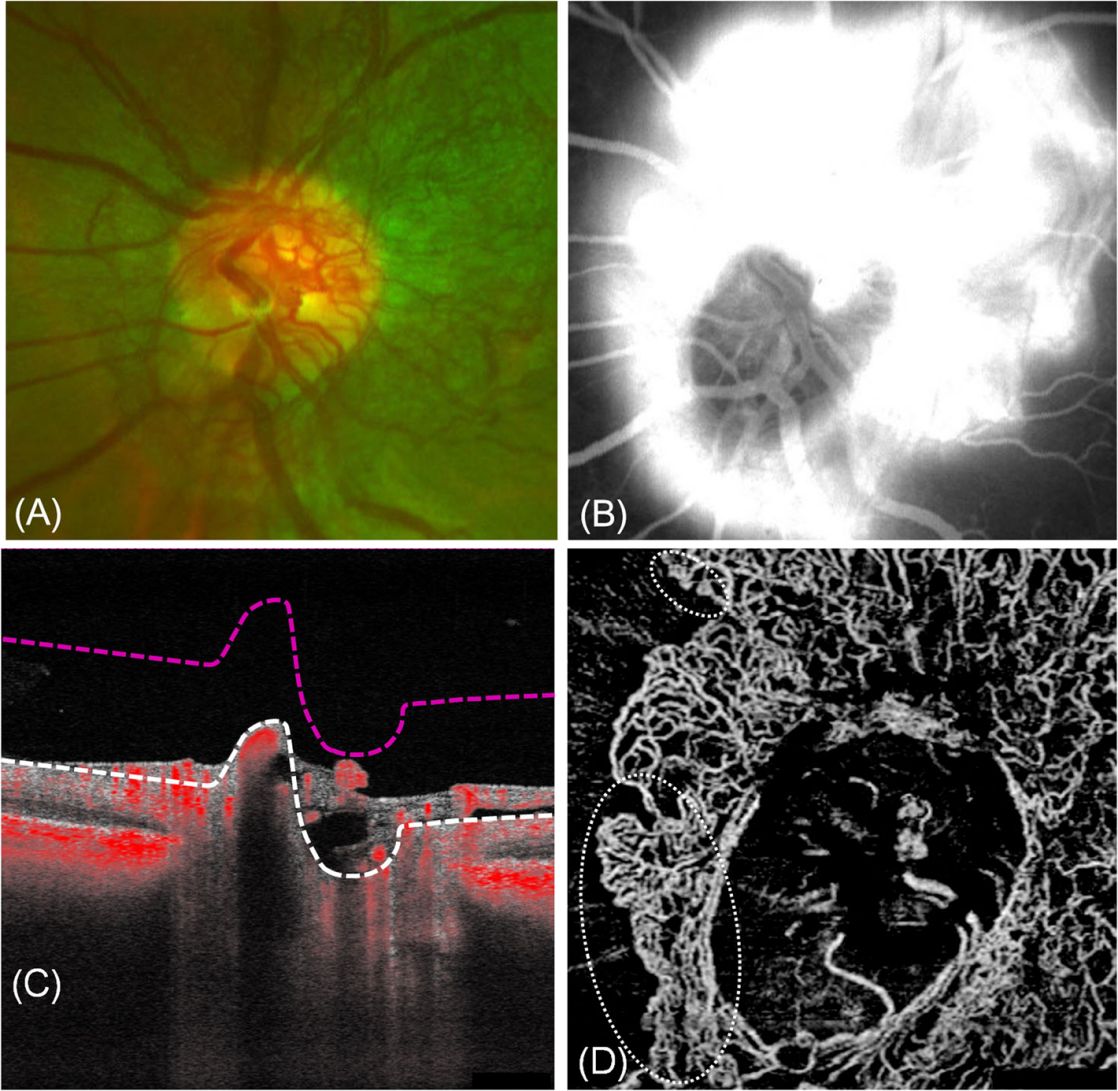
Neovascularization of the disc. **a** Fundus photography revealed the randomly oriented vessels over the optic disc. **b** Fluorescein angiography showed a profound early hyperfluorescence by fluorescein leaks from the new vessels. **c** The B-scan image displayed flow signals above the vitreoretinal interface (VRI, white dashed line) (**d**) The correlating VRI slab (between white and red dashed line in **c**) image depicted signal flows of the randomly oriented new vessels over the optic disc. Exuberant vascular proliferations are observed at the margin of NVD (ellipse). (The images depicted here are unrelated to the present case. Used with due patient consent and with approval of the ethics committee)

The vascular configuration of NVD is typically randomly oriented and usually shows more profound leakage in the early phase of fluorescein angiography (FA) than DP, but these findings are not much helpful for the differential diagnosis. NVD is observed as preretinal proliferative structure or protrusion into the vitreous cavity on optical coherence tomography (OCT) B-scan image [4]. On OCT angiography (OCTA), NVD displays positive flow signals elevated above the vitreoretinal interface (VRI) [5]. The correlating VRI slab image depicts signal flows of the randomly oriented new vessels. Exuberant vascular proliferations (EVPs), which are the intense growth of irregular small-caliber vessels and likely represent active proliferation [6], are observed at the margins of NVD (ellipse) (Fig. 1).

In the management of diabetic patient, DP must be distinguished from NVD which suggests high-risk proliferative diabetic retinopathy (PDR) that requires intensive intervention. However, differentiating DP from NVD is a diagnostic challenge. Herein, we present a case of DP in which swept-source optical coherence tomography angiography (SS-OCTA) was utilized to differentiate DP from NVD, which in turn greatly affected the treatment strategy.

## Case presentation

A 52-year-old man was referred for evaluation of an optic disc abnormality. He was on a regimen of oral anti-hypertensive agents and had no other known medical disease. At the initial presentation, his blood pressure was 133/86 mmHg.

**Fig. 2:**
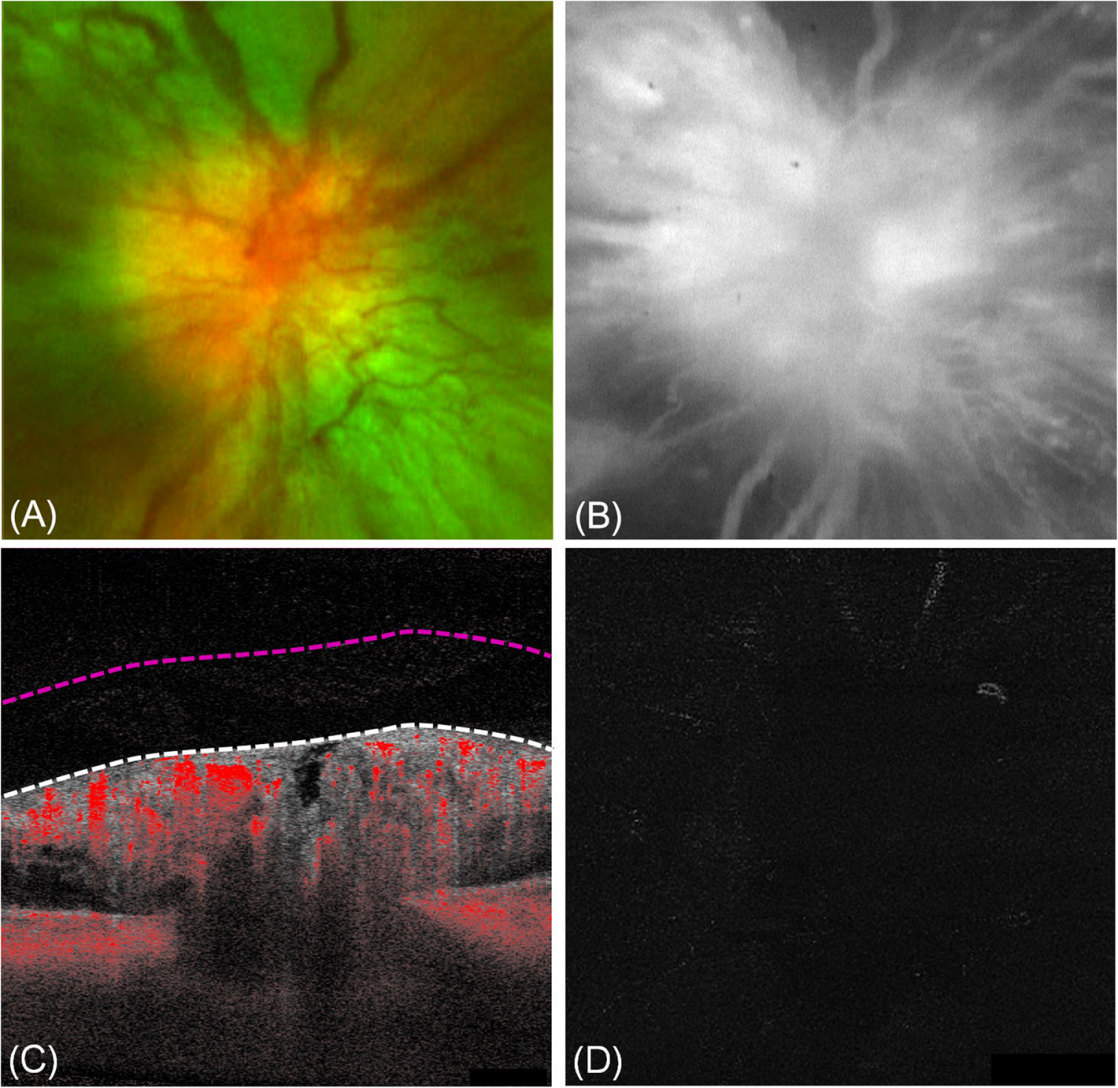
Diabetic papillopathy. **a** Fundus photography revealed a swollen optic disc, splinter hemorrhages, and dilated vessels over the optic disc. **b** Fluorescein angiography showed an early hyperfluorescence by fluorescein leaks from the disc vessels. **c** The B-scan image displayed blood flow signals in the thickened retinal nerve fiber layer of the optic disc and no flow signal above the vitreoretinal interface (VRI, white dashed line). **d** The correlating VRI slab (between white and red dashed line in **c**) image did not depict signal flow

His best-corrected visual acuity (BCVA) was 20/40 in the right eye and 20/100 in the left eye. Fundus examination demonstrated a swollen optic disc, splinter hemorrhages, and radially oriented, dilated vessels over the optic disc in both eyes (Fig. 2a). Microaneurysms and dot-blot hemorrhages were also noted in the mid-peripheral retina, but no signs of hypertensive retinopathy were observed. FA showed an early hyperfluorescence that increased throughout the study, most likely due to telangiectasia of the optic disc (Fig. 2b). Humphrey central 30–2 visual field test showed normal findings in the right eye and mid-peripheral depression with high false negative rate in the left eye. Pattern visual evoked potential showed normal findings in both eyes. Clinical neurologic examinations and brain magnetic resonance imaging revealed no abnormal findings. Laboratory tests, including markers for inflammation, infection, and connective tissue disease, showed no abnormalities other than an elevated blood glucose of 217 mg per deciliter and glycosylated hemoglobin level of 10.2%.

**Fig. 3:**
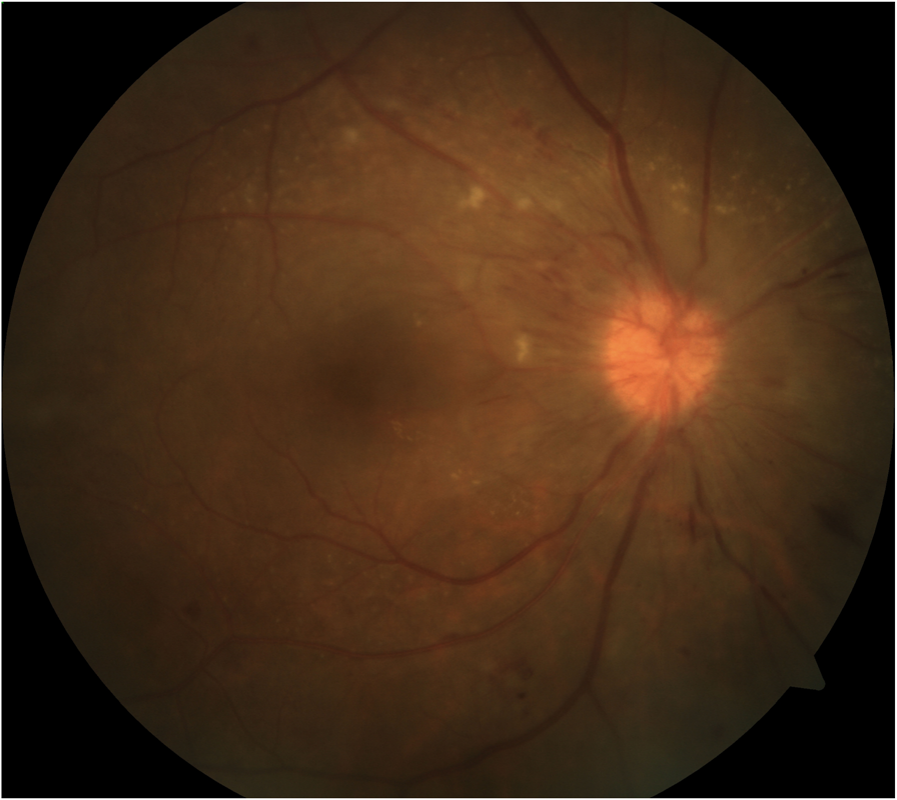
Resolving diabetic papillopathy. One month after the initial presentation, disc swelling and telangiectasia decreased only by blood glucose control without ophthalmic treatment

This patient was newly diagnosed with DM and diabetic retinopathy of both eyes. To rule out NVD which suggests high-risk PDR, SS-OCTA (PLEX Elite 9000; Carl Zeiss Meditec, Inc., Dublin, CA, USA) was performed. The B-scan image displayed blood flow signals in the thickened retinal nerve fiber layer of the optic disc and not above the VRI (white dashed line) (Fig. 2c), and the correlating VRI slab (between white and red dashed line in Fig. 2c) image did not depict signal flow (Fig. 2d), which suggested the presence of DP rather than NVD. The patient was managed with blood glucose control and close ophthalmologic follow-ups. One month later, optic disc swelling and telangiectasia had decreased (Fig. 3), and his final BCVA was 20/40 in both the eyes.

## Discussion and conclusions

The Diabetic Retinopathy Study (DRS) revealed that the presence and extent of NVD had the strongest association with severe visual loss (SVL) [7]. In the DRS, any NVD associated vitreous or preretinal hemorrhage, or NVD ≥1/3–1/4 disc area was classified into the high-risk PDR group, which had significantly greater risk of SVL and demonstrated the greatest benefit from pan-retinal photocoagulation [8]. This suggests that prompt detection of NVD is crucial in the management of patients with diabetic retinopathy. In contrast, serial examination remains the most common line of management for DP because of the self-limiting nature of this condition [9]. Thus, distinction between DP and NVD has a significant impact on the treatment strategy.

However, DP is often misdiagnosed as NVD because of the similar morphology of disc vessel changes and the early hyperfluorescence on FA. Currently, FA is the gold standard for examination of the vessels and the detection of retinal neovascularization, which is depicted as a remarkable dye leakage towards the vitreous cavity. However, FA is usually not helpful for distinguishing DP from NVD, because telangiectatic vessels of DP and NVD both display early hyperfluorescence due to dye leakage, which can obscure the vascular details of the pathologic vessel changes.

OCTA has several advantages over FA in that it is non-invasive and faster. OCTA offers better clarity, quantitative measurements, and three-dimensional visualization of the retinal microvasculature [10]. OCTA is a suitable technique to distinguish DP from NVD as it is unaffected by fluorescein leakage and can offer a clear image of the vascular details, like EVPs. In addition, OCTA also offers cross-sectional imaging which allows demarcation of the position of the microvasculature change above or below the VRI. The fine and radially oriented telangiectatic vessels in DP can be clearly visualized using OCTA and its location below VRI can also be confirmed. This is the first report that highlights that DP can be differentiated from NVD using SS-OCTA, which can be easily utilized in the clinical setting. In conclusion, SS-OCTA is useful for distinguishing DP from NVD based on clear visualization of microvasculature changes and whether flow signal lesions are observed above VRI or on the VRI slab. This method can be used to quickly plan the treatment strategy without additional invasive tests, such as FA.

## Data Availability

All data generated or analysed during this study are included in this published article.

## Abbreviations

DP: Diabetic papillopathy
DM: Diabetes mellitus
NVD: Neovascularization of the disc
PDR: Proliferative diabetic retinopathy
SS-OCTA: Swept-source optical coherence tomography angiography
FA: Fluorescein angiography
VRI: Vitreoretinal interface
